# Investigation on the hesitancy of COVID-19 vaccination among liver transplant recipients: A cross-sectional study in China

**DOI:** 10.3389/fpubh.2022.1014942

**Published:** 2022-12-15

**Authors:** Yixiao Pan, Shiming Gong, Xinye Zhu, Chuqing Xue, Yilei Jing, Yinghua Sun, Yongbing Qian, Jianjun Zhang, Qiang Xia

**Affiliations:** ^1^Department of Liver Surgery, Renji Hospital, School of Medicine, Shanghai Jiao Tong University, Shanghai, China; ^2^Department of Endocrinology, Children's Hospital School of Medicine, Zhejiang University, Hangzhou, China

**Keywords:** COVID-19, prevention, vaccine survey, vaccine hesitancy, vaccine acceptance, liver transplantation

## Abstract

**Objectives:**

The hospitalization and mortality rate from COVID-19 appears to be higher in liver transplant recipients when compared with general populations. Vaccination is an effective strategy to reduce the risk during the COVID-19 pandemic. We aimed to evaluate COVID-19 vaccine hesitancy in liver transplant recipients.

**Methods:**

In April 2022, we conducted an online-based survey through WeChat platform to investigate the vaccination hesitancy among liver transplant recipients followed at Shanghai Renji Hospital and further explore possible influencing factors. Survey items included multiple choice, Likert-type rating scale and open-ended answers. Participants were classified as no hesitancy group and hesitancy group. Using univariate analysis, ROC curve analysis and multiple logistic regression to evaluate associations between baseline characteristics and COVID-19 vaccine hesitancy.

**Results:**

449 liver transplant recipients participated in the survey with 299 (66.6%) of them being categorized as vaccine hesitancy. In no hesitancy group, 73 (48.7%) recipients had completed vaccination, while 77 (51.3%) were not yet but intended to be vaccinated. In contrast, 195 (65.2%) recipients in hesitancy group were hesitant to get vaccinated, while the remaining 104 (34.8%) refused. The most common side effect was injection arm pain (*n* = 9, 12.3%). The common reasons for vaccine willingness was trusted in the effectiveness of the vaccine and fear of contracting COVID-19. The most common reason for vaccination hesitancy is fear of side effects, and the most effective improvement was the support from the attending physician. Factors associated with vaccine hesitancy include female sex, influenza vaccination status, awareness of the importance and safety of vaccine, attitudes of doctors and others toward vaccine, medical worker source information of vaccine, relative/friend with medical background, total score of VHS (Vaccine Hesitancy Scale), accessibility of vaccine.

**Conclusion:**

For liver transplant recipients, COVID-19 vaccine is an important preventive measure. Identifying the factors influencing COVID-19 vaccine hesitancy is therefore critical to developing a promotion plan. Our study shows that more comprehensive vaccine knowledge popularization and relevant medical workers' training can effectively improve the acceptance of COVID-19 vaccine in this population.

## Introduction

In December 2019, COVID-19 has caused a pandemic in many countries around the world. In March 2022, Omicron, a mutated COVID-19 virus, began to spread in China, especially Shanghai, causing a major blow to economy, medical system, and social life. Compared with the previously detected COVID-19 virus, this variant is more infectious and poses a serious threat to the health of vulnerable populations (e.g., the elderly, hematology patients, solid organ transplant recipients). Solid organ transplant recipients (e.g., liver) appear to be more susceptible to COVID-19 and have higher rates of hospitalization and mortality compared with other populations due to large immunosuppressants after surgery and potential comorbidities (1, 2). The mortality rate among solid organ transplant recipients infected with COVID-19 has been reported between 13 and 30% (1). Safe and effective vaccines are essential to reduce the risk of COVID-19, protect vulnerable populations, and prevent the pandemic. Currently, more than 280 COVID-19 vaccines are in development, and many of them have entered the Chinese healthcare system, such as Sinovac and Sinopharm (3, 4).

At the end of March 2021, the National Health Commission of the People's Republic of China released the first edition of COVID-19 vaccine vaccination technical guideline to further popularize and promote vaccination, but it lacked detailed description of solid organ transplant recipients (4). While some other guidelines [e.g., AISF (5), EASL (6), and AASLD (7)] strongly recommend that liver transplant recipients should be vaccinated against COVID-19. However, one of the major obstacles to promote COVID-19 vaccination is vaccine hesitancy (8). According to the World Health Organization (WHO), vaccination hesitancy means the delay in acceptance or reluctance of vaccination despite availability of vaccination services, which has been recognized as one of the 10 threats to global health due to the declining vaccination rates (9).

According to several online questionnaires, solid organ transplant recipients' vaccine hesitancy about COVID-19 was mainly attributed to concerns about its side effects, potential comorbidities, and doctors' negative advice (10, 11). Several secondary factors were also associated with vaccine hesitancy, including type of graft, main source of vaccine information, education level, influenza vaccination experience and willingness, perceptions of the importance of COVID-19 vaccines, risk perception and trust, and religious and moral beliefs (8). Other unreported factors may also be involved, such as the surprising speed of COVID-19 vaccine development, the relatively lack of efficacy and safety data in solid organ transplant recipients (6, 12–14), and the spread and amplification of negative information about vaccines by some organization or individual (15).

Current surveys of COVID-19 vaccine hesitancy have focused on health workers, students, patients with chronic diseases, the elderly, and children, and have rarely included solid organ transplant recipients. We reviewed the literature and found small number of reports on the willingness of liver transplant recipients to be vaccinated against COVID-19 (11). It has reported that solid organ transplant recipients are generally associated with low willingness to get vaccinated against COVID-19. However, the majority of these subjects were kidney transplant recipients (10). So far, there has been no related investigation about immunosuppressed people after liver transplantation in China. To fill this gap, we conducted such a survey to identify factors influencing vaccine hesitancy among liver transplant recipients in China and to promote vaccine promotion.

## Materials and methods

### Study design and sample

An anonymous, self-designed, and structured online questionnaire was conducted in Chinese liver transplant recipients aged 18 years and above, from 26 April to 10 May 2022. The questionnaire was made available through WeChat platform, released by the department of Liver Surgery, Renji Hospital Shanghai Jiao Tong University. A web link collector generated the survey QR code through which participants could access the survey and send their answers. Inclusion criteria included: adult recipients (age ≥18 years old) who were followed up after liver transplantation in our hospital. Exclusion criteria included: pre-transplant vaccination against COVID-19, missing or illogical questionnaire information, loss of follow-up. Ethical approval was granted by the Ethics Committee of Renji Hospital Shanghai Jiaotong University (No. KY2022-138-B). Participants in this study were voluntary, and an informed consent was placed at the top of the questionnaire. Patients who give consent to inform will access to the subsequent questionnaire. Completion of the anonymous survey did not result in any benefit or financial compensation for the recipients. The confidentiality of all data was guaranteed (ClinicalTrials.gov Identifier: NCT05532592). Participants were classified as no hesitancy group (NHG) and hesitancy group (HG) to accept COVID-19 vaccination. COVID-19 vaccines were totally free in China and offered independently of the questionnaire responses.

### Survey items

**Figure 1:**
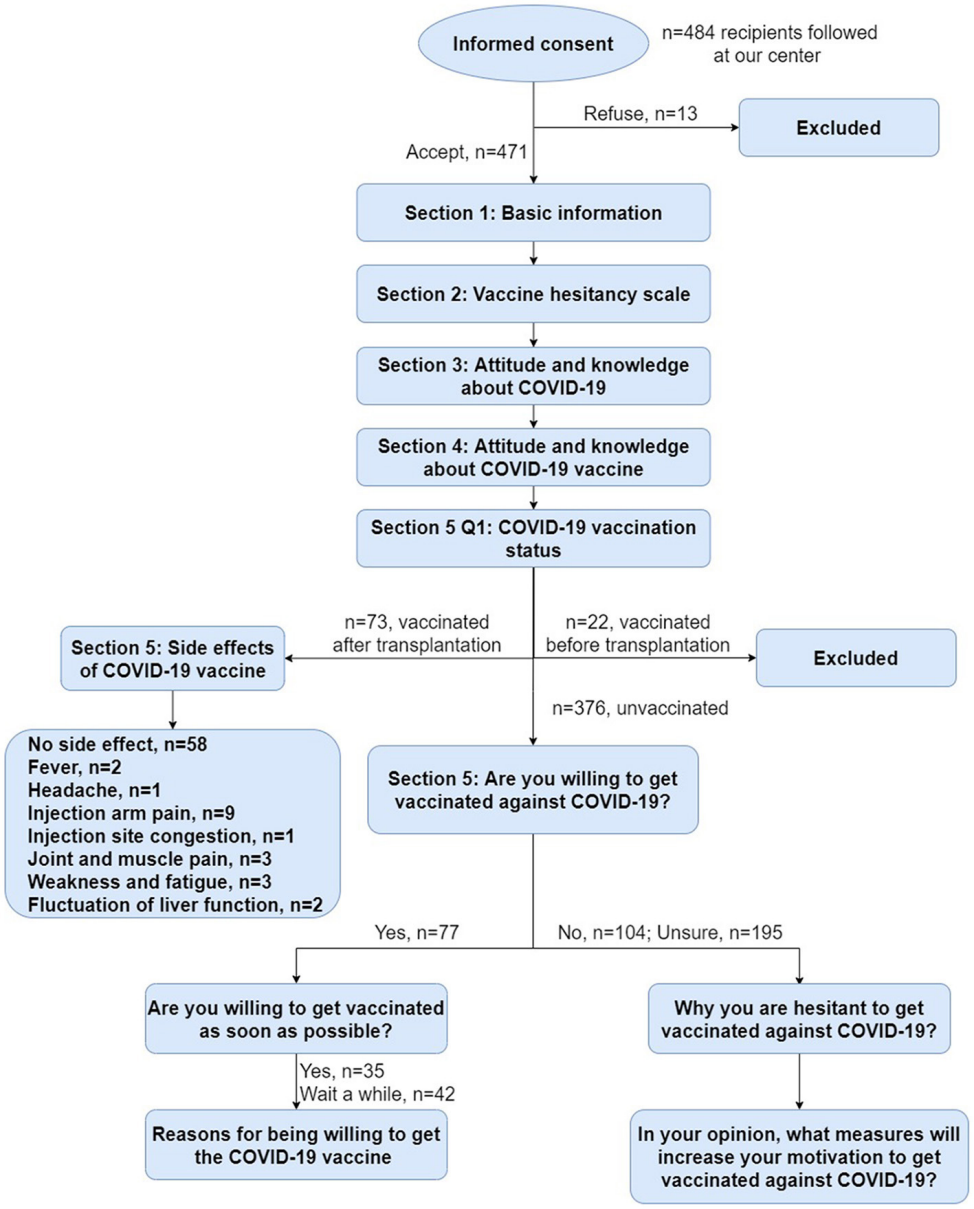
The flow chart for the five sections of the questionnaire.

Our follow-up questionnaire comprised five sections (Figure 1). For details of the questionnaire items, please refer to the corresponding table or the supplementary materials we have uploaded. The first section includes demographic data, health state, transplantation and medication, chronic diseases and allergy history, influenza vaccination. The second section is a scale (VHS) to quantify vaccination hesitancy among liver transplant recipients. The third section is about the attitudes and perceptions of the participants toward COVID-19. The fourth section investigates the knowledge of the participants about COVID-19 vaccines. The final section confirms their vaccination status and evaluates their vaccine acceptance or hesitancy.

Vaccine Hesitancy Scale (VHS) was developed by the WHO SAGE Working Group on Vaccine Hesitancy that was widely used in different countries and settings (16–18). VHS comprised 10 items about adult attitudes toward vaccination and each item was scored 10–50 and summed to calculate a total score, with higher score indicating greater hesitancy. In this study, we used the 10 items of the VHS that are measured on a five-point Likert-type rating scale ranging from “strongly agree” to “strongly disagree.” No changes were made to the wording of the items. We administered questions in a random order to mitigate any order effect. We reversed three items in the scoring of the scale so that higher scores indicated more hesitancy on all items. The survey items are available at: https://doi.org/10.6084/m9.figshare.13207145

### Statistical analysis

Statistical analysis was performed using IBM SPSS 23.0. Categorical variables were presented as number (percentage), and quantitative variables were presented as mean ± standard deviation. Chi-square test was used for univariate analysis of categorical variables. Student's *t*-test were used for quantitative variables. Mann-Whitney *U*-test were used for ranking variables. Variables with *p* < 0.1 in the univariate analysis were included in multiple logistic regression analysis, to assess factors associated with vaccination hesitancy. Odds ratio and 95% confidence intervals were calculated. The Hosmer–Lemeshow test and Omnibus test were performed for the model fit estimation. ROC curve analysis was used to calculate the cutoff point of VHS results. The level of statistical significance was set at *p* < 0.05.

## Results

### Demographic data and sample characteristics

Overall, 484 recipients from follow-up list participated in the online survey between 26 April and 10 May 2022. A total of 471 valid questionnaires were obtained. The response rate was 97.3%. Among these participants, 22 recipients received COVID-19 vaccine before transplantation, so we excluded them. Finally, 449 recipients met the criteria for inclusion in this study.

Based on the WHO definition of vaccine hesitancy mentioned above, we considered that there was no vaccine hesitancy in recipients who got vaccine after transplantation or were willing to be vaccinated. Therefore, we classified them into the no hesitancy group (NHG). Accordingly, recipients who were uncertain or rejective, were identified as vaccine hesitancy, and we categorize them into the hesitancy group (HG).

Subsequently, a total of 150 recipients were enrolled in the NHG (Vaccinated/Willing to be vaccinated), including 73 (48.7%) recipients vaccinated after liver transplantation and 77 (51.3%) who were currently unvaccinated but willing to be vaccinated. And there were 299 recipients in the HG (Unwilling or uncertain of vaccination), including 195 (65.2%) who were uncertain and 104 (34.8%) who refused vaccination.

Of the 449 recipients, male was the majority (*n* = 308, 68.6%), compared with 141 female (31.4%). Mean (±standard deviation) age was 54.56 (±10.69) years old, with most recipients located in the 45–60 age range. The primary etiology of transplantation was mainly hepatitis B (because only one case was hepatitis C) (*n* = 193, 43%), followed by autoimmune liver disease (*n* = 119, 26.5%), liver tumor (*n* = 60, 13.4%), and others (*n* = 77, 17.1%). Most recipients reported to have exceeded 12 months after transplantation, with a mean time of 64.67 months. All respondent recipients were adhering to their immunosuppressive therapy and most of them had regular follow-up biopsy (*n* = 366, 81.5%). Other related information and significant difference between the two groups are shown in Table 1.

**Table 1:** Demographic data and sample characteristics of participants.

		**Variables**	**Total participants**	**Vaccination status**		*p*-Value^*^
			**(*n*, %)**	**Vaccinated/willing to be vaccinated (*n*, %)**	**Unwilling or uncertain of vaccination (*n*, %)**	
		Total (*n*)	449	150	299	
Sex		Male	308 (68.6)	115 (76.7)	193 (64.5)	0.009^*^
		Female	141 (31.4)	35 (23.3)	106 (35.5)	
Age (years)		>18 and ≤30	10 (2.2)	2 (1.3)	8 (2.7)	0.49
		>30 and ≤45	71 (15.8)	26 (17.3)	45 (15.1)	
		>45 and ≤60	248 (55.2)	87 (58)	161 (53.8)	
		>60	120 (26.7)	35 (23.3)	85 (28.4)	
		Age (mean ± standard deviation)	54.56 ± 10.694	54.85 ± 10.61	54.41 ± 10.751	0.682
Nationality		Han	434 (96.7)	145 (96.7)	289 (96.7)	0.995
		Others	15 (3.3)	5 (3.3)	10 (3.3)	
Marital status		Married	401 (89.3)	135 (90)	266 (89)	0.737
		Single/divorced/widowed	48 (10.7)	15 (10)	33 (11)	
Occupation		Enterprise workers	135 (30.1)	50 (33.3)	85 (28.4)	0.042^*^
		Farmer	35 (7.8)	10 (6.7)	25 (8.4)	
		Government officers	36 (8)	15 (10)	21 (7)	
		Retired/vacation^#^	182 (40.5)	47 (31.3)	135 (45.2)	
		Student	8 (1.8)	3 (2)	5 (1.7)	
		Other vocations^#^	53 (11.8)	25 (16.7)	28 (9.4)	
Living situation		Live alone	41 (9.1)	15 (10)	26 (8.7)	0.651
		With family	408 (90.9)	135 (90)	273 (91.3)	
Residence		Urban	365 (81.3)	119 (79.3)	246 (82.3)	0.451
		Rural	84 (18.7)	31 (20.7)	53 (17.7)	
Education level		High school or below	222 (49.4)	72 (48)	150 (50.2)	0.665
		College or above	227 (50.6)	78 (52)	149 (49.8)	
Monthly income per capita (RMB)		>20,000	57 (12.7)	22 (14.7)	35 (11.7)	0.313
		10,000–20,000	94 (20.9)	37 (24.7)	57 (19.1)	
		5,000–10,000	170 (37.9)	54 (36)	116 (38.8)	
		<5,000	128 (28.5)	37 (24.7)	91 (30.4)	
Have relative/friend with medical background		Yes	173 (38.5)	49 (32.7)	124 (41.5)	0.071
		No	276 (61.5)	101 (67.3)	175 (58.5)	
Self-assessment of health		I'm healthy	206 (45.9)	80 (53.3)	126 (42.1)	0.025^*^
		Uncertain or unhealthy	243 (54.1)	70 (46.7)	173 (57.9)	
Causes of transplantation		Hepatitis B or C^#^	193 (43)	79 (52.7)	114 (38.1)	0.019^*^
		Autoimmune liver disease (including PBC/PSC)^#^	119 (26.5)	29 (19.3)	90 (30.1)	
		Liver tumor	60 (13.4)	17 (11.3)	43 (14.4)	
		Others	77 (17.1)	25 (16.7)	52 (17.4)	
Post-transplantation time		≤3 months	9 (2)	3 (2)	6 (2)	0.177
		>3 and ≤6 months	14 (3.1)	1 (0.7)	13 (4.3)	
		>6 and ≤12 months	36 (8)	11 (7.3)	25 (8.4)	
		>12 months	390 (86.9)	135 (90)	255 (85.3)	
		Time (mean ± standard deviation)	64.67 ± 54.147	66.42 ± 54.328	63.79 ± 54.126	0.627
Type of immunosuppressant used^a^		1	184 (41)	70 (46.7)	114 (38.1)	0.104
		2	202 (45)	65 (43.3)	137 (45.8)	
		≥3	63 (14)	15 (10)	48 (16.1)	
Immunological rejection by biopsy		Yes	73 (16.3)	24 (16)	49 (16.4)	0.755
		No	293 (65.3)	101 (67.3)	192 (64.2)	
		Uncertain due to no biopsy	83 (18.5)	25 (16.7)	58 (19.4)	
Treatment of primary disease		Cure	399 (88.9)	143 (95.3)	256 (85.6)	0.002^*^
	Not healed	50 (11.1)	7 (4.7)	43 (14.4)		
Chronic disease	Endocrine diseases	Yes	147 (32.7)	49 (32.7)	98 (32.8)	0.981
		No	302 (67.3)	101 (67.3)	201 (67.2)	
	Chronic respiratory diseases	Yes	30 (6.7)	11 (7.3)	19 (6.4)	0.695
		No	419 (93.3)	139 (92.7)	280 (93.6)	
	Cardiovascular and cerebrovascular diseases	Yes	137 (30.5)	54 (36)	83 (27.8)	0.074
		No	312 (69.5)	96 (64)	216 (72.2)	
	Chronic nephrosis	Yes	39 (8.7)	16 (10.7)	23 (7.7)	0.291
		No	410 (91.3)	134 (89.3)	276 (92.3)	
	Chronic liver diseases	Yes	55 (12.2)	15 (10)	40 (13.4)	0.303
		No	394 (87.8)	135 (90)	259 (86.6)	
	Immune system diseases	Yes	4 (0.9)	0 (0)	3 (1)	0.537
		No	445 (99.1)	150 (100)	296 (99)	
	Tumor	Yes	14 (3.1)	2 (1.3)	12 (4)	0.21
		No	435 (96.9)	148 (98.7)	287 (96)	
	Others	Yes	177 (39.4)	57 (38)	120 (40.1)	0.663
		No	272 (60.6)	93 (62)	179 (59.9)	
HBV+ now		Yes	91 (20.3)	23 (15.3)	68 (22.7)	0.065
		No	358 (79.7)	127 (84.7)	231 (77.3)	
Drug allergy history		Yes	72 (16)	16 (10.7)	56 (18.7)	0.028^*^
		No	377 (84)	134 (89.3)	243 (81.3)	
Food allergy history		Yes	19 (4.2)	2 (1.3)	17 (5.7)	0.031^*^
		No	430 (95.8)	148 (98.7)	282 (94.3)	
Vaccine allergy history		Yes	8 (1.8)	5 (3.3)	3 (1)	0.167
		No	441 (98.2)	145 (96.7)	296 (99)	
Delay or refuse vaccinations except for illnesses or allergies		Yes	101 (22.5)	21 (14)	80 (26.8)	0.002^*^
		No	348 (77.5)	129 (86)	219 (73.2)	
Influenza vaccination during last year (2021–2022)		Yes	16 (3.6)	15 (10)	1 (0.3)	0.001^*^
		No	433 (96.4)	135 (90)	298 (99.7)	
Intention toward influenza vaccination for the current season		Yes	31 (6.9)	24 (16)	7 (2.3)	0.001^*^
		No	418 (93.1)	126 (84)	292 (97.7)	
Other vaccines after transplantation (except for influenza and		Yes	33 (7.3)	14 (9.3)	19 (6.4)	0.254
COVID-19)		No	416 (92.7)	136 (90.7)	280 (93.6)	

a Immunosuppressants including: tachlimus, mycophenolate mofetil, mycophenolate sodi, prednisone, rapamycin, cyclosporine.

* p-values <0.05 are marked with an asterisk.

# Subgroups with differences in univariate analysis.

### Vaccine hesitancy scale

Vaccine hesitancy scale scores of the two groups were displayed in Table 2. Mann-Whitney *U*-test for each item score and Student's *t*-test for the total score showed significant differences and HG scored significantly higher than NHG, suggesting that HG had a significantly higher quantification of vaccine hesitancy on the scale.

**Table 2:** Vaccine hesitancy scale result.

**Items**	**Vaccination status**		*p*-Value^*^
	**Vaccinated/willing to be vaccinated (mean ±standard deviation)**	**Unwilling or uncertain of vaccination (mean ±standard deviation)**	
Vaccines are important for my health	17.87 ± 10.781	27.32 ± 13.837	0.001^*^
Vaccines are effective	18.07 ± 10.146	26.45 ± 13.291	0.001^*^
Being vaccinated is important for the health of others in my community.	15.67 ± 9.653	21.1 ± 12.815	0.001^*^
All routine vaccinations recommended by the CDC are beneficial	18.07 ± 10.911	22.27 ± 12.458	0.001^*^
New vaccines carry more risks than older vaccines.	26.6 ± 14.601	29.26 ± 13.138	0.047^*^
The information I receive about vaccines from the CDC is reliable and trustworthy.	19 ± 10.666	24.01 ± 12.149	0.001^*^
Getting vaccines is a good way to protect me from disease.	16.67 ± 10.144	22.24 ± 12.638	0.001^*^
Generally, I do what my doctor or healthcare provider recommends about vaccines for me.	14.8 ± 10.148	17.79 ± 11.577	0.002^*^
I am concerned about serious adverse effects of vaccines.	32.2 ± 14.346	39.36 ± 13.334	0.001^*^
I do not need vaccines for diseases that are not common anymore.	28 ± 15.801	32.21 ± 13.968	0.006^*^
Total score	207.93 ± 75.559	262.04 ± 78.091	0.001^*^

* p-values <0.05 are marked with an asterisk.

**Figure 2:**
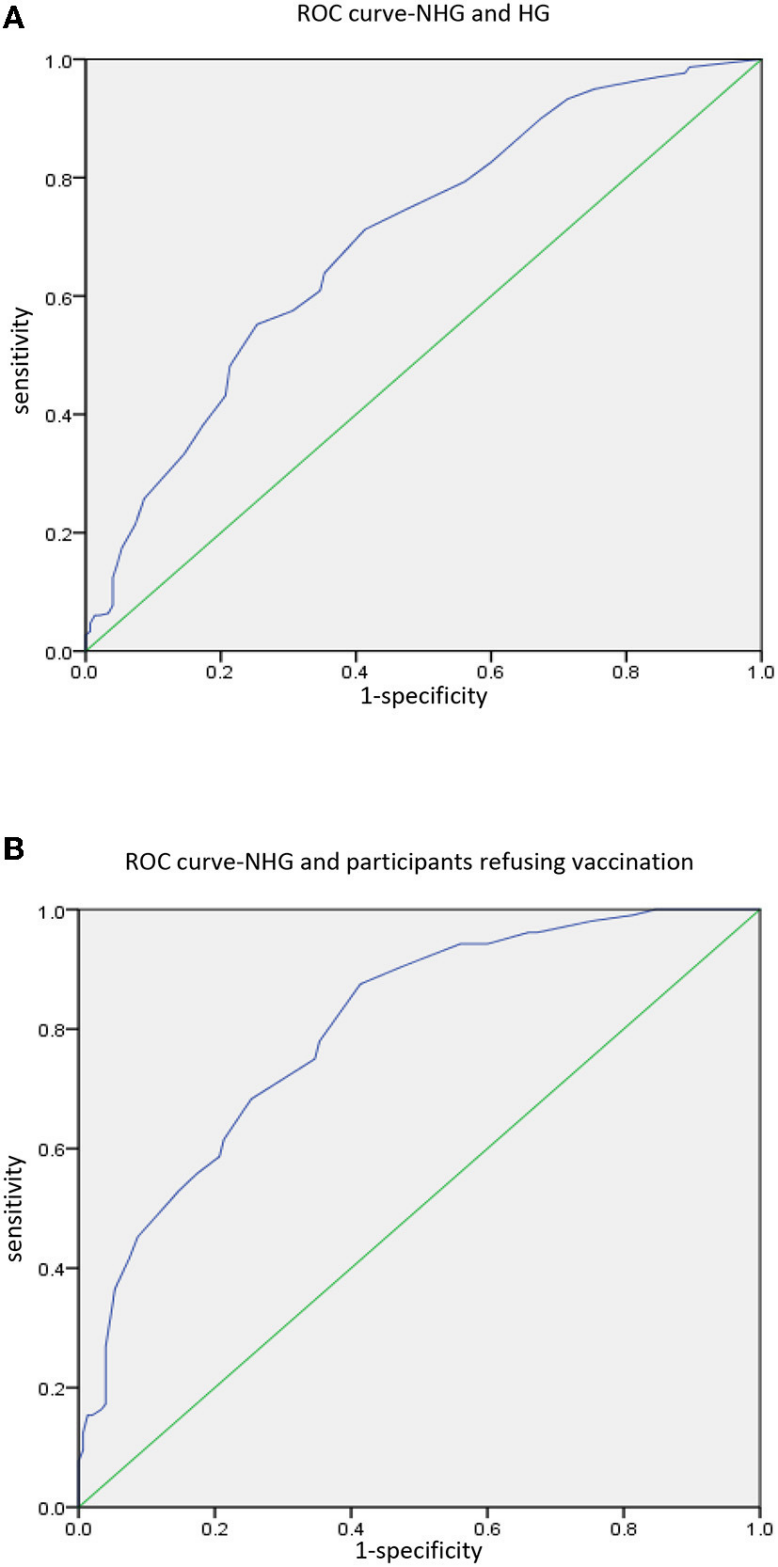
ROC curve analysis. **(A)** Analysis between NHG and HG. **(B)** Analysis between NHG and participants refusing vaccination.

Then we conducted ROC curve analysis for NHG and HG, and NHG and participants refusing vaccination, to calculate the cutoff point of VHS results (Figure 2). The results of the ROC curve analysis are shown in Table 3. The cutoff point between NHG and HG was 215 (*p* < 0.001), with the sensitivity 71.2%, and the specificity 58.7%. While the cutoff point between NHG and participants refusing vaccination was also 215 (*p* < 0.001), with the sensitivity 87.5%, and the specificity 58.7%.

**Table 3:** ROC curve analysis results of vaccine hesitancy scale.

	**AUC**	**95% CI**	*p*-Value^*^	**Cutoff point**	**Sensitivity**	**Specificity**
ROC curve analysis for NHG and HG	0.696	0.644–0.747	0.001^*^	215	71.2%	58.7%
ROC curve analysis for NHG and participants refusing vaccination	0.802	0.749–0.856	0.001^*^	215	87.5%	58.7%

AUC, area under the curve; 95% CI, 95% confidence interval; NHG, no hesitancy group; NH, hesitancy group.

* p-values <0.05 are marked with an asterisk.

### Attitude toward COVID-19

Attitudes and perceptions of COVID-19 were almost identical between the two groups and significant differences only existed in two items (Table 4). We can see that NHG participants learn about COVID-19 more thoroughly [yes vs. no: 51 (34%) vs. 66 (22.1%); *p* = 0.007], and their occupational risk of COVID-19 was relatively higher [high vs. low risk: 63 (42%) vs. 94 (31.4%); *p* = 0.027].

**Table 4:** Results of attitude toward COVID-19.

		**Variables**	**Total participants**	**Vaccination status**		*p*-Value^*^
			**(*n*, %)**	**Vaccinated/willing to be vaccinated (*n*, %)**	**Unwilling or uncertain of vaccination (*n*, %)**	
		Total (*n*)	449	150	299	
Know COVID-19^a^		Yes	117 (26.1)	51 (34)	66 (22.1)	0.007^*^
		Uncertain or no	332 (73.9)	99 (66)	233 (77.9)	
Have you ever had COVID-19		Yes	5 (1.1)	4 (2.7)	1 (0.3)	0.081
		No	444 (98.9)	146 (97.3)	298 (99.7)	
Are you worried about getting COVID-19 (first or again)		Yes	399 (88.9)	130 (86.7)	269 (90)	0.294
		No	50 (11.1)	20 (13.3)	30 (10)	
Do you have any friends or family members who have had COVID-19		Yes	45 (10)	14 (9.3)	31 (10.4)	0.731
		No	404 (90)	136 (90.7)	268 (89.6)	
Occupational risk of COVID-19		High	157 (35)	63 (42)	94 (31.4)	0.027^*^
		Low	292 (65)	87 (58)	205 (68.6)	
Risk of COVID-19 infection in patients after liver transplantation		Higher	276 (61.5)	97 (64.7)	179 (59.9)	0.342
		General or lower	74 (16.5)	26 (17.3)	48 (16.1)	
		Uncertain	99 (22)	27 (18)	72 (24.1)	
Impact of COVID-19 on the health of patients after liver transplantation		More serious	343 (76.4)	119 (79.3)	224 (74.9)	0.484
		General or less	32 (7.1)	8 (5.3)	24 (8)	
		Uncertain	74 (16.5)	23 (15.3)	51 (17.1)	
What worries you as a liver transplant patient about the current COVID-19	Infection leads to recurrence of the disease or interfere with recovery	Yes	376 (83.7)	121 (80.7)	255 (85.3)	0.211
pandemic		No	73 (16.3)	29 (19.3)	44 (14.7)	
	The symptoms and consequences of COVID-19 are more serious	Yes	291 (64.8)	103 (68.7)	188 (62.9)	0.226
		No	158 (35.2)	47 (31.3)	111 (37.1)	
	Hospital or community control leads to drug dispensing difficulties	Yes	275 (61.2)	91 (60.7)	184 (61.5)	0.858
		No	174 (38.8)	59 (39.3)	115 (38.5)	
	Affect access to health care	Yes	267 (59.5)	85 (56.7)	182 (60.9)	0.392
		No	182 (40.5)	65 (43.3)	117 (39.1)	
	Increase the cost of treatment	Yes	144 (32.1)	40 (26.7)	104 (34.8)	0.082
		No	305 (67.9)	110 (73.3)	195 (65.2)	
	Other worries	Yes	30 (6.7)	9 (6)	21 (7)	0.682
		No	419 (93.3)	141 (94)	278 (93)	

a Know COVID-19: have some knowledge of the prevention measures, symptoms, prognosis and treatment of COVID-19.

* p-values <0.05 are marked with an asterisk.

### Knowledge about COVID-19 vaccine

In this section, when comparing COVID-19 vaccine hesitancy or not, there were apparent differences between the two groups, including: awareness of the first edition of COVID-19 vaccine vaccination technical guideline, awareness of the side effects and precautions of COVID-19 vaccine, the main source of information on COVID-19 vaccine, safety of COVID-19 vaccine, efficacy of COVID-19 vaccine, whether vaccination can help control the epidemic and promote the health of society, COVID-19 vaccine will lead to the recurrence of the primary disease, COVID-19 vaccine is not safe for post-transplantation, COVID-19 vaccine is inconvenient for post-transplantation, safety of COVID-19 vaccine in post-transplantation, efficacy of COVID-19 vaccine in post-transplantation, COVID-19 vaccine is important for liver transplant patients, have actively sought advice about COVID-19 vaccine, surgery doctor's attitude toward COVID-19 vaccine, family and friends' attitudes toward COVID-19 vaccine. Detailed information is shown in Table 5. These differences are in line with our expectations. Overall, recipients in NHG were more knowledgeable about the COVID-19 vaccine, had more trust in the vaccine, and received more support. We will continue our analysis as followings.

**Table 5:** Results of knowledgement about COVID-19 vaccine.

		**Variables**	**Total participants**	**Vaccination status**		*p*-Value^*^
			**(*n*, %)**	**Vaccinated/willing to be vaccinated (*n*, %)**	**Unwilling or uncertain of vaccination (*n*, %)**	
		Total (*n*)	449	150	299	
Know the first edition of COVID-19 vaccine vaccination technical guideline		Yes	121 (26.9)	52 (34.7)	69 (23.1)	0.009^*^
		No	328 (73.1)	98 (65.3)	230 (76.9)	
Know the side effects and precautions of COVID-19 vaccine		Yes	133 (29.6)	56 (37.3)	77 (25.8)	0.011^*^
		No	316 (70.4)	94 (62.7)	222 (74.2)	
The main source of information on COVID-19 vaccine		Social media platforms^#^	214 (47.7)	83 (55.3)	131 (43.8)	0.012^*^
		TV programs and news releases	94 (20.9)	28 (18.7)	66 (22.1)	
		Family, friends or community	93 (20.7)	33 (22)	60 (20.1)	
		Medical worker^#^	31 (6.9)	4 (2.7)	27 (9)	
		Others	17 (3.8)	2 (1.3)	15 (5)	
Safety of COVID-19 vaccine		Safe	117 (26.1)	62 (41.3)	55 (18.4)	0.001^*^
		Not safe or uncertain	332 (73.9)	88 (58.7)	244 (81.6)	
Efficacy of COVID-19 vaccine		Yes	210 (46.8)	90 (60)	120 (40.1)	0.001^*^
		No or uncertain	239 (53.2)	60 (40)	179 (59.9)	
Vaccination helps control the epidemic and the health of society		Agree	289 (64.4)	115 (76.7)	174 (58.2)	0.001^*^
		Disagree/uncertain	160 (35.6)	35 (23.3)	125 (41.8)	
Availability of COVID-19 vaccine		Convenient	422 (94)	143 (95.3)	279 (93.3)	0.395
		Inconvenient	27 (6)	7 (4.7)	20 (6.7)	
As a liver transplant patient, what are your main concerns about getting the	Affect recovery after liver transplantation	Yes	227 (50.6)	71 (47.3)	156 (52.2)	0.333
COVID-19 vaccine		No	222 (49.4)	79 (52.7)	143 (47.8)	
	Lead to the recurrence of the primary disease	Yes	189 (42.1)	49 (32.7)	140 (46.8)	0.004^*^
		No	260 (57.9)	101 (67.3)	159 (53.2)	
	Affect post-transplant medication	Yes	223 (49.7)	68 (45.3)	155 (51.8)	0.193
		No	226 (50.3)	82 (54.7)	144 (48.2)	
	More serious side effects	Yes	321 (71.5)	101 (67.3)	220 (73.6)	0.167
		No	128 (28.5)	49 (32.7)	79 (26.4)	
	Not safe for post-transplantation	Yes	258 (57.5)	64 (42.7)	194 (64.9)	0.001^*^
		No	191 (42.5)	86 (57.3)	105 (35.1)	
	Doubtful validity for post-transplantation	Yes	192 (42.8)	55 (36.7)	137 (45.8)	0.064
		No	257 (57.2)	95 (63.3)	162 (54.2)	
	Inconvenient for post-transplantation	Yes	195 (43.4)	38 (25.3)	157 (52.5)	0.001^*^
		No	254 (56.6)	112 (74.7)	142 (47.5)	
	Other concerns	Yes	13 (2.9)	5 (3.3)	8 (2.7)	0.925
		No	436 (97.1)	145 (96.7)	291 (97.3)	
Safety of COVID-19 vaccine in post-transplantation		Safe	55 (12.2)	49 (32.7)	6 (2)	0.001^*^
		Not safe or uncertain	394 (87.8)	101 (67.3)	293 (98)	
Efficacy of COVID-19 vaccine in post-transplantation		Yes	75 (16.7)	51 (34)	24 (8)	0.001^*^
		No or uncertain	374 (83.3)	99 (66)	275 (92)	
COVID-19 vaccine is important for liver transplant patients		Agree	159 (35.4)	97 (64.7)	62 (20.7)	0.001^*^
		Disagree/uncertain	290 (64.6)	53 (35.3)	237 (79.3)	
Have actively sought advice about COVID-19 vaccine		Yes	318 (70.8)	121 (80.7)	197 (65.9)	0.001^*^
		No	131 (29.2)	29 (19.3)	102 (34.1)	
Surgery doctor's attitude toward COVID-19 vaccine		Support	100 (22.3)	65 (43.3)	35 (11.7)	0.001^*^
		Neutral or rejective	349 (77.7)	85 (56.7)	264 (88.3)	
Family and friends' attitudes toward COVID-19 vaccine		Support	203 (45.2)	98 (65.3)	105 (35.1)	0.001^*^
		Neutral or rejective	246 (54.8)	52 (34.7)	194 (64.9)	
Family members have got COVID-19 vaccine		Yes	438 (97.6)	149 (99.3)	289 (96.7)	0.159
		No	11 (2.4)	1 (0.7)	10 (3.3)	

* p-values <0.05 are marked with an asterisk.

# Subgroups with differences in univariate analysis.

### Vaccination status

(1) Recipients vaccinated after surgery: side effects

**Figure 3:**
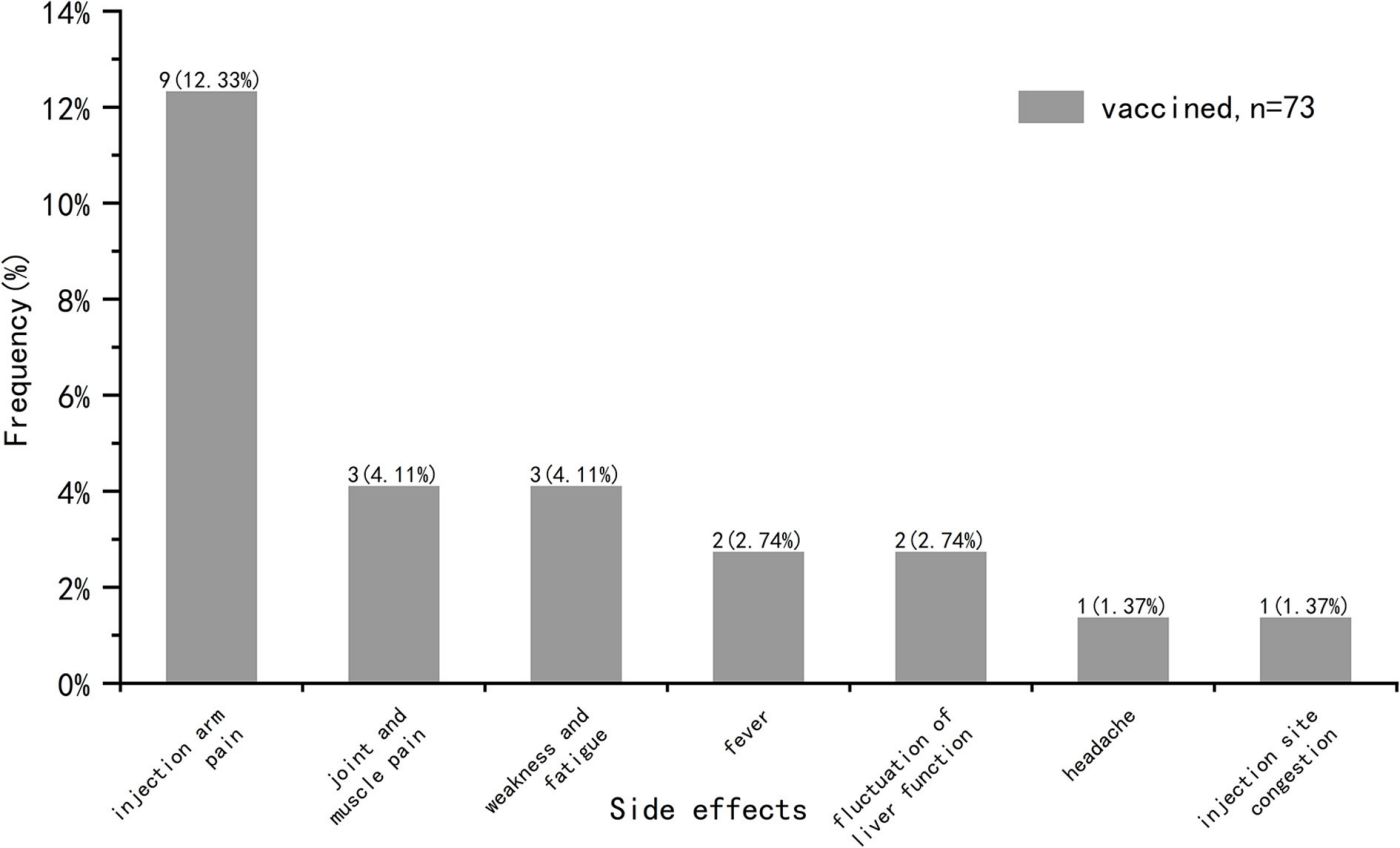
COVID-19 vaccine related side effects in vaccinated participants.

We analyzed common vaccine-related side effects in post-transplantation vaccinated COVID-19 recipients (n = 73), including fever, headache, tinnitus, light-headed, injection arm pain, injection site congestion, numbness of the arm, joint and muscle pain, weakness and fatigue, sore throat, nausea/vomiting, diarrhea, skin rash, anaphylaxis, edema, hypertensive attack, heart-related side effects, fluctuation of liver function, other side effects (Figure 3). These symptoms were self-reported by participants and not diagnosed by medical institutions. It was found that the incidence of side effects in our study was 20.55%, and there was no symptom serious or requiring medical attention. The most common side effect was injection arm pain, followed by joint and muscle pain, weakness and fatigue, fever, fluctuation of liver function, injection site congestion and headache.

(2) Unvaccinated but willing to be vaccinated: reasons for willing to get vaccinated

**Figure 4:**
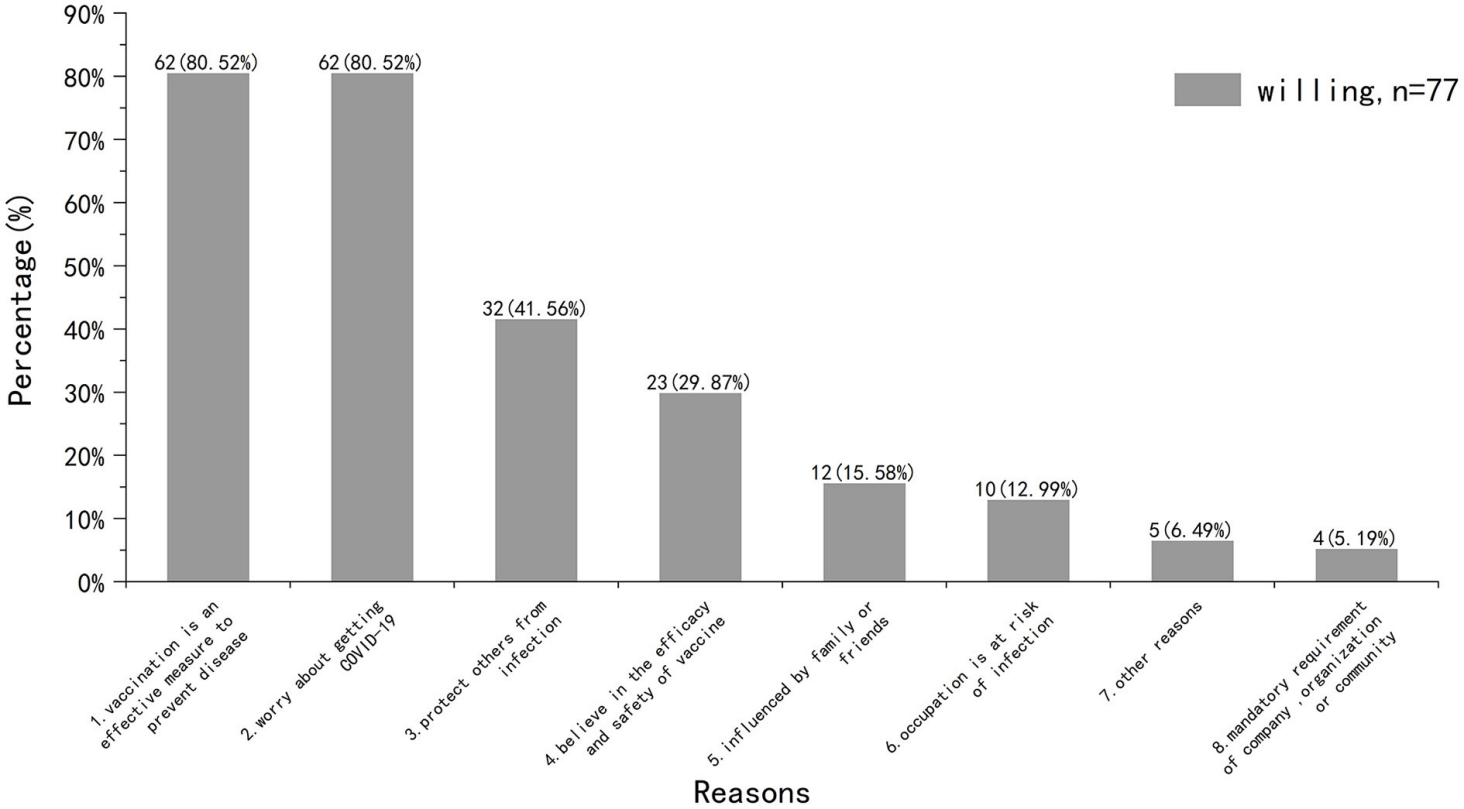
Reasons for willing to get vaccinated.

We then surveyed participants who were willing to be vaccinated (Figure 4). Half of the participants (n = 35, 45.45%) were willing to get vaccinated as soon as possible, while the other half (n = 42, 54.55%) wanted to wait a while. As for the reasons for willing to get vaccinated, the highest proportion were “vaccination is an effective measure to prevent disease (n = 62, 80.52%)” and “worry about getting COVID-19 (n = 62, 80.52%).”

(3) Unvaccinated but hesitant or refusing to be vaccinated: reasons for vaccination hesitancy and management

**Figure 5:**
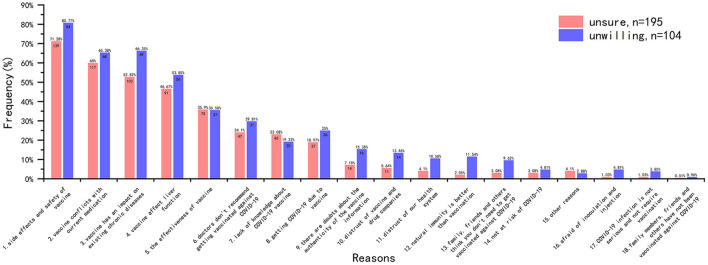
Reasons for vaccination hesitancy.

The main reasons for vaccination hesitancy were analyzed from the data of the 299 participants in HG (Figure 5). The results showed that among these participants who were unsure to be vaccinated (*n* = 195, 65.22%), the most common reason was “side effects and safety of vaccine (*n* = 139, 71.28%),” followed by “vaccine conflicts with current medication (*n* = 117, 60.00%),” “vaccine has an impact on existing chronic diseases (*n* = 103, 52.82%)” and “vaccine affect liver function (*n* = 91, 46.67%).” The main reasons for the participants unwilling to be vaccinated (*n* = 104, 34.78%) were also the same with different order.

**Figure 6:**
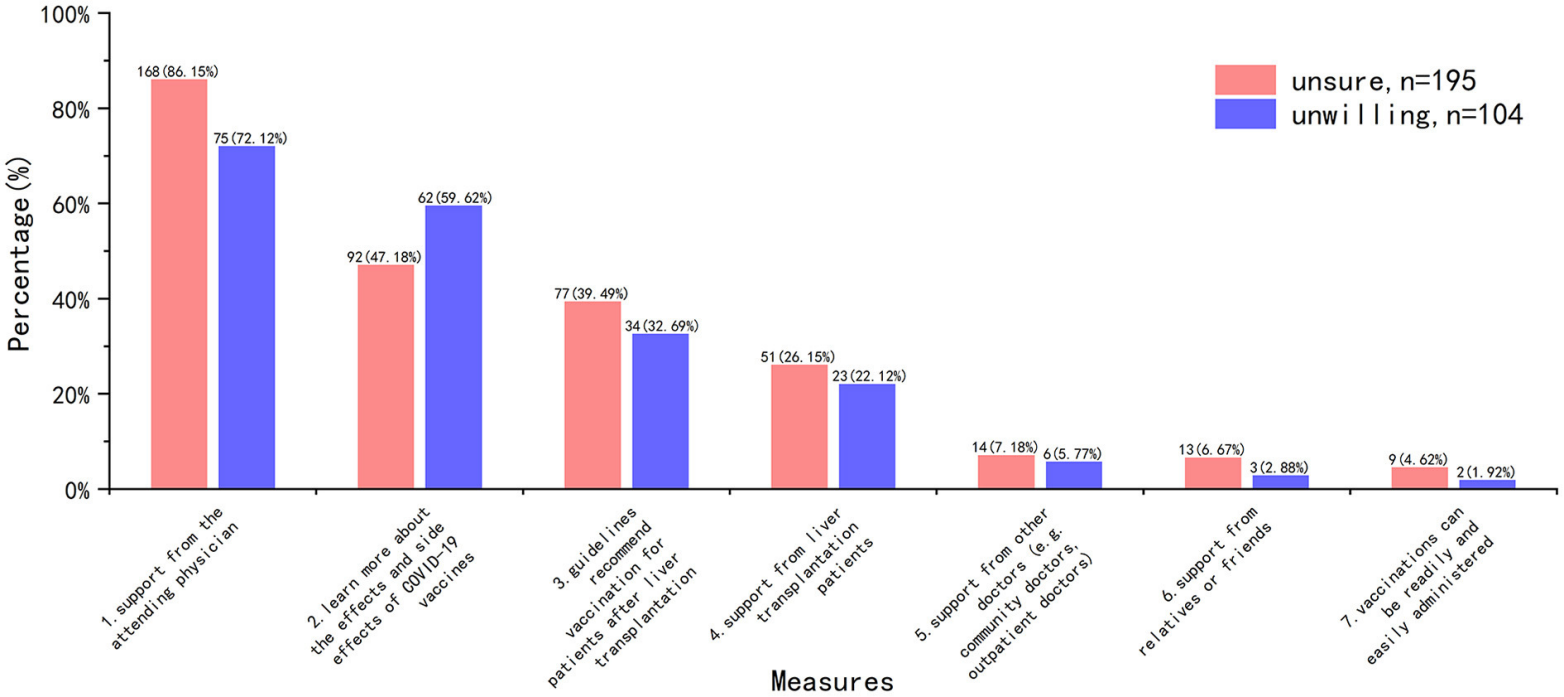
Measures to improve COVID-19 vaccine hesitancy.

Measures to improve COVID-19 vaccine hesitancy were consistent, with the overwhelming majority of HG recipients opting for the support of their attending physician (Figure 6).

### Logistics regression results: Predictors for vaccine hesitancy

**Figure 7:**
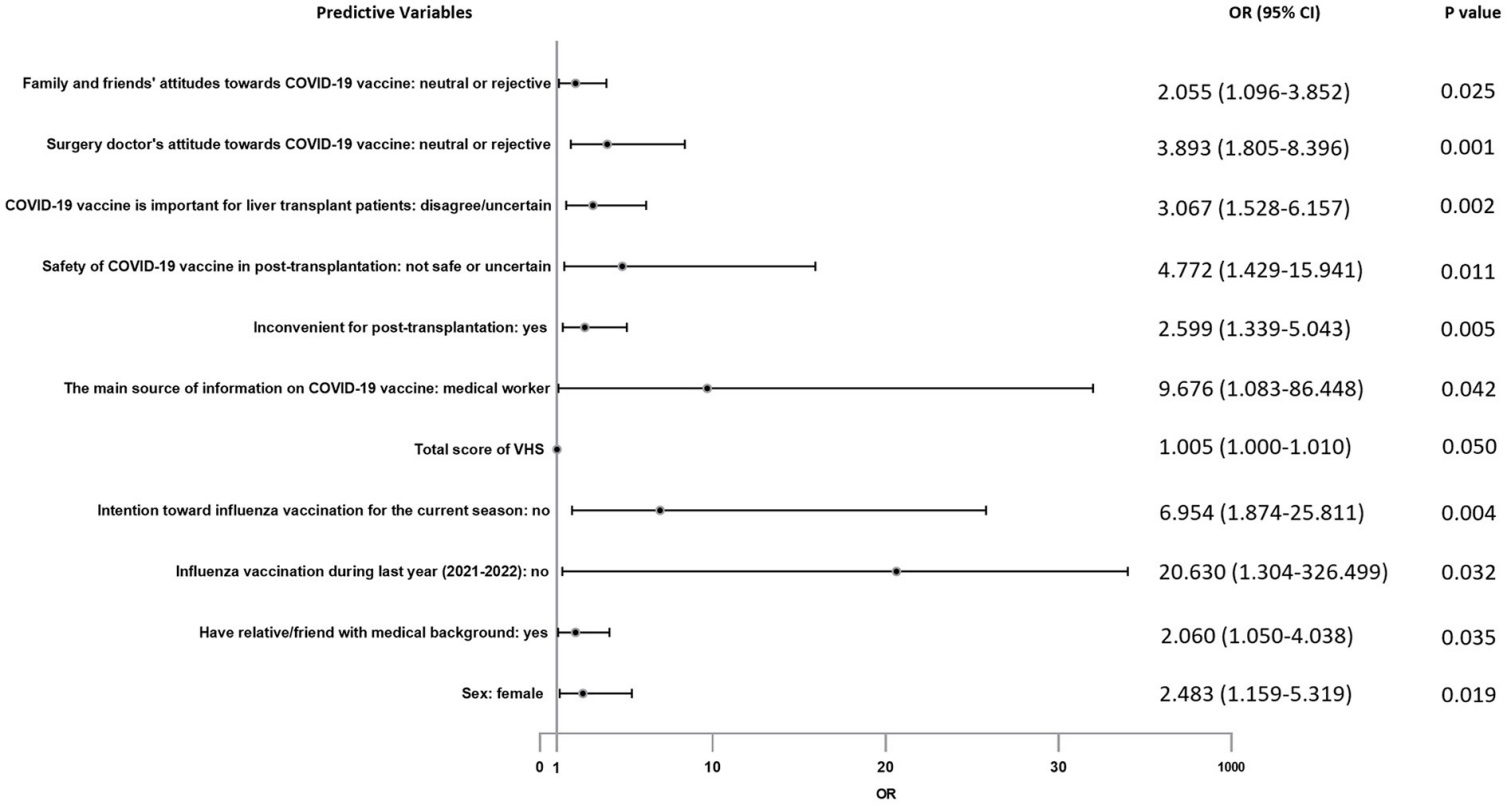
Forest plot of predictors for vaccine hesitancy.

For the above items with statistical results *p* < 0.1, they were included in multiple logistic regression analysis to further explore their correlation with vaccine hesitancy. In the logistic regression analysis result as showed in Figure 7, factors positively associated with vaccination hesitancy are followings: female recipients (OR = 2.483, 95% CI = 1.159–5.319), had relative/friend with medical background (OR = 2.060, 95% CI = 1.050–4.038), refused to get influenza vaccination during last year (2021–2022) (OR = 20.630, 95% CI = 1.304–326.499), had no intention toward influenza vaccination for the current season (OR = 6.954, 95% CI = 1.874–25.811), total score of VHS (OR = 1.005, 95% CI = 1.000–1.010), the main source of information on COVID-19 vaccine was medical worker (OR = 9.676, 95% CI = 1.083–86.448), COVID-19 vaccination is inconvenient for post-transplantation (OR = 2.599, 95% CI = 1.339–5.043), distrusted the safety of COVID-19 vaccine in post-transplantation (OR = 4.772, 95% CI = 1.429–15.941), not perceived the importance of COVID-19 vaccine for liver transplant patients (OR = 3.067, 95% CI = 1.528–6.157), surgery doctor did not recommend COVID-19 vaccination (OR = 3.893, 95% CI = 1.805–8.396), family or friends believed they should not get COVID-19 vaccine (OR = 2.055, 95% CI = 1.096–3.852).

## Discussion

Coronavirus is derived from the Latin word “corona” meaning “crown”(19). It causes a range of human respiratory tract infections varying from mild cold to severe respiratory distress syndrome (20). The present coronavirus disease 2019 (COVID-19) is an emerging global health threat. It is known to be acquired from a zoonotic source and typically spreads through contact and droplet transmission (21). It started from Wuhan city of China at the end of December 2019 and since then spread rapidly around the world, creating a pandemic.

Nowadays, COVID-19 vaccination is considered to be the most appropriate measure to prevent COVID-19 infection, reduce the severity caused by COVID-19 infection and control COVID-19 pandemic. So far, the Chinese government and communities have made great efforts to promote the nationwide vaccination against COVID-19, including but not limited to publishing the first edition guideline of COVID-19 vaccine vaccination (4), popularization of COVID-19 vaccine on social networks and other platforms, completely free COVID-19 vaccine and even certain material rewards to encourage vaccination. A global survey of COVID-19 vaccines has revealed that Chinese residents have the highest acceptance (90%) (22). So far, China has made remarkable progress against COVID-19 compared to other regions, keeping the morbidity and mortality to a minimum, in which vaccines play an essential role.

However, while China's COVID-19 vaccine guideline recommends vaccination for immunocompromised people, including liver transplant recipients, there is no detailed description or data on the efficacy and safety of the vaccine in this population. As a result, liver transplant recipients are often hesitant to respond to government calls.

During routine follow-up after liver transplantation, we learned that some recipients had been vaccinated against COVID-19, while most were on the sidelines. Therefore, we hope to explore the common causes and influencing factors of vaccine hesitancy among liver transplant recipients through this survey. A previous survey among Chinese solid organ transplant recipients showed insufficient vaccination rate and willingness, most commonly due to fear of comorbidities (10). Associated factors included type of transplantation organ, the main source of vaccine information, education level, influenza vaccination intention, influenza vaccination status in the previous season, and perception of the importance of vaccines.

Unfortunately, the majority of study involved kidney transplant recipients, but only very few liver transplant recipients. Due to the differences in surgical methods, post-operative immunosuppressive usage, and many other aspects, it may be difficult to directly apply the information of kidney transplant recipients to liver transplant recipients. There are also differences in time and social environment: this study started in June 2021, when the epidemic situation in China was relatively stable, the time of COVID-19 vaccines introduction in China was relatively short, and there was a lack of information on the use COVID-19 vaccines in immunosuppressed population. Our study was carried out in May 2022, when China was facing a severe epidemic, especially in Shanghai, a medical and economic hub, where the omicron variant was rampant, and the safety and effectiveness of COVID-19 vaccine in transplant recipients were confirmed (12, 13).

Our results are partly in line with expectations and explain their vaccine hesitancy. In our study, there were 150 participants (33.4%) who were willing or completed vaccination after transplantation, and 299 participants (66.6%) with vaccine hesitancy who were unwilling or uncertain about vaccination. Although there was still a gap between this result and that of normal adults in China (60.4–82.3%) (23–26) or liver transplant recipients in Italy (85.3%) (11), we believe there had been a significant improvement compared to previous survey (10). Due to the limited literature on adult liver transplant recipients, it is difficult to compare our outcome with other regions or countries.

Among participants who had completed COVID-19 vaccination (*n* = 73), the incidence of side effects was 20.55% and the most common reported symptom was pain at the injection arm. This was in line with the results reported by Boyarsky et al. (27) and Erol et al. (28). Among participants who were willing to be vaccinated (*n* = 77), about half of them (*n* = 35, 45.45%) wanted to be vaccinated as soon as possible, while the rest (*n* = 42, 54.55%) wanted to wait a while. Previous study attributed this delay to distrust in the efficacy and safety of the vaccine (11). We believe that the higher proportion of delayed vaccination in our study may be due to the severity of the epidemic in China during the investigation period, the high risk of COVID-19 transmission and the closed-loop management measures in some areas. Also, we consider the main reasons for COVID-19 vaccine willingness mentioned above are related to this situation.

Why did participants hesitate to get the COVID-19 vaccine? We investigated major factors of vaccine hesitancy in HG. Concerns about vaccine safety and side effects were the most common reason among participants who were unsure or refused to receive the vaccine. Several other reasons that were relatively common (close to 50% or above) included “vaccine has an impact on existing chronic diseases,” “vaccine conflicts with current medication” and “vaccine affect liver function,” which were about fear of comorbidities or the impact on graft. This result is highly similar to that of Costantino et al. (11) and Ou et al. (29), suggesting that side effects, transplant organ, and comorbidities are the main factors that cause vaccine hesitancy in related population.

When we asked what improvements were needed in HG to increase their willingness to get COVID-19 vaccine, a noticeable finding was that “support from the attending physician” topped significantly the other choices (*n* = 243, 81.27%), while only 6.69% HG participants reported “support from other doctors” was helpful. However, we noted that 88.3% (*n* = 264) of the HG participants reported that their surgery doctors had “neutral or rejective” attitude, but only 26.09% (*n* = 78) of them had vaccine hesitancy due to “doctors don't recommend getting vaccinated against COVID-19.” Therefore, it was not difficult to assume that most attending physician's response to the recipient's post-transplantation vaccination was equivocal. Recipients trust their attending physician, and doctors' “hesitancy” will contribute to their “vaccine hesitancy.” Aslam et al. (30) reported that experience with influenza and zoster vaccines in solid organ transplant population can be applied to COVID-19 vaccine. In fact, some liver associations or organizations have published guidelines or recommendations on COVID-19 vaccination for liver transplant recipients (5–7). These literatures suggest that transplant recipients are at a higher risk of poor prognosis, and COVID-19 vaccine is recommended early after transplantation. In addition, some studies have reported good safety in liver transplant and other solid organ transplant recipients after receiving COVID-19 vaccine (12, 13). Even though efficacy may be insufficient (low antibody levels), this is not a reason to deny preventative protection. However, according to our survey, even attending physicians, let alone other non-transplant physicians, have limited knowledge about COVID-19 vaccine. Therefore, in order to effectively improve the willingness of transplant recipients to COVID-19 vaccine, it is necessary to strengthen the training of doctors, especially attending doctors, or publish relevant popular science articles on the basis of hospitals/departments.

We assessed factors associated with vaccine hesitancy in terms of four sections mentioned above. In the initial univariate analysis (Chi-square test), many significant differences were found between NHG and HG. After multiple logistic regression analysis and excluding confounding factors, our results showed that some factors were related to vaccine hesitancy independently. It is surprising that women are more likely to be reluctant to get vaccinated. Similar studies rarely yield differences between the sexes. We have two hypotheses for this: women think more about pain, side effects or other vaccine-related factors; our study was not a random sample, which may be due to sampling bias. We were shocked that the main source of vaccine information from medical workers was a contributing factor to vaccine hesitancy. This result was in stark contrast to several previous studies (10). Subsequently, we conducted a one-to-one telephone follow-up of these HG participants (*n* = 27) and learned that all the suggestions given by medical workers were uncertain or opposed. Therefore, the essence of this phenomenon was medical workers had limited COVID-19 vaccine knowledge, and we speculated that “have relative/friend with medical background contributing to vaccine hesitancy” was also related to this. Earnshaw et al. (31) highlighted doctors as the most trusted source of information about COVID-19. Doctor's advice greatly influences patient's behavior. So that was why a neutral or negative recommendation from their surgery doctor would cause obvious vaccine hesitancy. Of course, neutral/negative advice from family and friends also played a role. Consistent with studies conducted by Gan et al. (23) and Alfageeh et al. (32), people without influenza vaccination during last year (2021–2022) were more hesitant to be vaccinated. Similarly, people with negative intention toward influenza vaccination for the current season were more likely to have vaccine hesitancy. Garcia et al. and Di Gennaro et al. (33, 34) came to the same conclusion. Our survey indicated that people who denied the importance and safety of vaccines for liver transplant recipients had more hesitancy. They believe that vaccination might be harmful to them and would not protect them from COVID-19. It suggested that improving patients' knowledge of vaccine will help to increase the vaccine willingness. As for “inconvenient for post-transplantation,” it is easy to understand that the nationwide containment management has caused a lot of inconvenience, including medical activity.

Vaccine hesitancy scale is an effective tool for investigating vaccine hesitancy in adults. In our study, Student's *t*-test, ROC curve analysis and logistics regression all proved that participants with higher total score were more hesitant to get vaccinated. As mentioned above, we conducted ROC curve analysis twice among different populations, and the cutoff points obtained were all 215. This result is similar to general population survey conducted by Akel et al. (16). In this regard, we believe that VHS can be used as a tool for mass screening during follow-up of liver transplant recipients to identify potential recipients with COVID-19 vaccine hesitancy and give them appropriate relative advice.

Of course, there are many limitations in our results, which may cause some bias. First, the participants were not randomly sampled. Instead, we gave questionnaires based on WeChat platform to liver transplant recipients in follow-up and they voluntarily chose to participate or not. This may lead to selection bias and exclude some potential participants who had difficulty answering questionnaires online (such as the elderly, visual impairment, cognitive impairment, non-use of Internet/WeChat, etc.). Secondly, there were many items in our questionnaire (about 60 questions). Even if the respondents who agreed to participate in the survey were expected to fill in the questionnaire carefully and truthfully before the survey, there was still the possibility of being impatient or even filling in the questionnaire carelessly. Thirdly, compared with some other studies, our sample size was still insufficient. These defects should be avoided as much as possible in future studies.

## Conclusion

This is the first study to investigate the attitudes and hesitancy of liver transplant recipients toward vaccination after the introduction of COVID-19 vaccine in China. In summary, the continued hesitancy of liver transplant recipients to the COVID-19 vaccine is a hindrance to preventing the spread of COVID-19 in immunosuppressed population and controlling the epidemic. It is important to identify the factors that influence vaccine hesitancy in liver transplant recipients in order to establish appropriate improvements in doctor-patient communication. Our results listed possible related reasons and factors, highlighted the importance of more comprehensive vaccine health education, and emphasized the critical role of all health workers, including transplant physicians, in promoting vaccination. We hope our results will play a role in promoting vaccination campaigns for liver transplant recipients.

## Data availability statement

The raw data supporting the conclusions of this article will be made available by the authors, without undue reservation.

## Ethics statement

The studies involving human participants were reviewed and approved by Ethics Committee of Renji Hospital Shanghai Jiao Tong University (No. KY2022-138-B). The patients/participants provided their written informed consent to participate in this study.

## Author contributions

Conceived and designed the experiments and administrated the project: YP, YQ, and QX. Performed the data analysis: YP, SG, XZ, and CX. Validated the data: YP and XZ. Supervised the project: YQ, JZ, and QX. Wrote the paper: YP. All authors contributed to the article and approved the submitted version.
